# Enhanced induction of abnormal telomere FISH signals in response to oxidative DNA damage

**DOI:** 10.1093/jrr/rrad102

**Published:** 2024-01-03

**Authors:** Yoshimi Sakamoto, Kazunori Shiraishi, Seiji Kodama

**Affiliations:** Radiation Biology Group, Department of Biological Chemistry, Graduate School of Science, Osaka Metropolitan University, 1-2 Gakuen-cho, Naka-ku, Sakai, Osaka 599-8570, Japan; Radiation Biology Group, Department of Biological Chemistry, Graduate School of Science, Osaka Metropolitan University, 1-2 Gakuen-cho, Naka-ku, Sakai, Osaka 599-8570, Japan; Radiation Biology Group, Department of Biological Chemistry, Graduate School of Science, Osaka Metropolitan University, 1-2 Gakuen-cho, Naka-ku, Sakai, Osaka 599-8570, Japan

**Keywords:** telomere FISH signals, radicals, oxidative DNA damage, fluorescence *in situ* hybridization, DNA replication stress

## Abstract

Telomere dysfunction induces chromosomal instability, which is a driving force in the development of cancers. To examine X-irradiation’s effect on telomere integrity, we investigated X-ray-induced abnormalities in telomere signals detected by fluorescence *in situ* hybridization (telomere FISH) in mouse embryo fibroblast cells. The abnormalities were categorized as either extra telomere signals (ETSs) or loss of telomere signals (LTSs). The results indicated that low doses (0.3–0.5 Gy) of X-rays significantly induced ETS but not LTS and that ETS induction was saturated at doses above 0.5 Gy. In addition, treatment with hydrogen peroxide also induced ETS but not LTS. To clarify the involvement of radicals in inducing ETS, we examined the effect of ascorbic acid (AsA) on telomere FISH signals and found that pre-treatment with AsA (5 mM, 2 h), but not post-treatment, significantly suppressed the induction of ETS by X-irradiation. Importantly, neither pre- nor post-treatment with AsA affected X-ray-induced chromosome aberrations. These results suggest that oxidative DNA damage induced by radicals is involved in the induction of ETS. Furthermore, combined treatment with aphidicolin, a DNA replication inhibitor, elevated the induction of ETS by X-irradiation. This observation suggests that DNA replication stress, potentially triggered by oxidative DNA lesions within telomeres, may contribute to the induction of ETS resulting from X-irradiation. Based on these results, we propose that ETS is a sensitive biological marker of oxidative DNA damage in telomere structures.

## INTRODUCTION

In eukaryotic cells, linear chromosome ends need shielding from DNA damage surveillance mechanisms so that those mechanisms do not identify and treat them as DNA double strand breaks [1]. Telomeres are the ends of eukaryotic chromosomes, composed of tandem repeats of six bases of TTAGGG, and form loop structures together with shelterin to shield chromosome ends [2, 3]. Shelterin, a telomere-specific protein complex, consists of six proteins, Telomere Repeat binding Factor 1 (TRF1), Telomere Repeat binding Factor 2 (TRF2), Protection of Telomere 1 (POT1), Telomere Protection Protein 1 (TPP1), TRF1 and TRF2 interacting Nuclear Protein 2 (TIN2) and Repressor/Activator Protein 1 (RAP1), and represses the DNA damage response by preventing chromosome ends from being detected as DNA double strand breaks [1,4]. Guanine-rich telomeres are susceptible to oxidative stress because guanine has the lowest redox potential among the four DNA bases [5–8]. In addition, the fact that preferential accumulation of single-stranded regions was detected in telomeres following oxidative damage indicates that oxidative DNA damage tends to accumulate at telomeres [6]. Oxidative telomeres are more likely to induce telomere instability because of their reduced ability to bind to TRF1 and TRF2 [9]. Ionizing radiation generates intracellular radicals in exposed cells, and these radicals oxidize DNA. Thus, telomeres are assumed to be vulnerable to ionizing radiation. So far, however, there have been no sensitive and reliable biological markers to assess telomere instability.

Telomere repeats can be visualized by fluorescence *in situ* hybridization (FISH), specifically telomere FISH, which is targeted to the long arrays of telomere repeats. Several reports suggest the possibility that the telomere signal visualized by telomere FISH is an indicator of telomere integrity. Abnormal telomere FISH signals were induced in X-ray-surviving human diploid cells that had been immortalized by the *hTERT* gene [10], suggesting that aberrant telomere FISH signals are indicative of telomere instability persisting over several generations of X-irradiation. Furthermore, aberrant telomere FISH signals were reported to be elevated in cells that possess characteristics of telomere instability, such as Werner syndrome cells, *SIRT6*-depleted human cells, *Atm*-deficient mouse cells and *TRF1*-deficient mouse cells [11–16]. These results suggest that the abnormality of telomere FISH signals reflects defects in the structure and function of telomeres.

In the present study, we investigated abnormal telomere signals detected by telomere FISH as a quantitative indicator of telomere integrity by determining the effect of oxidative conditions induced by X-irradiation and hydrogen peroxide (H_2_O_2_). Our results indicate that abnormality of telomere signals is a sensitive marker of radiation exposure at low doses (0.3–0.5 Gy) and that radicals, which are removed by ascorbic acid (AsA), are involved in the generation of extra telomere signals (ETSs). We propose that, in combination with the enhanced effect of aphidicolin (Aph) on X-ray-induced abnormal telomere FISH signals, abnormality in telomere FISH signals serves as an indicator of oxidized telomere DNA, which might be a major cause of DNA replication stress.

## MATERIALS AND METHODS

### Cells and cell culture

Institute of Cancer Research (ICR) mouse embryos at 14.5 days of gestation were dissected, and the bodies, after removal of the heads, were finely chopped using scissors. The tissue pieces were then cultured in a T-25 (25 cm^2^) culture flask with 2 ml of minimum essential medium alpha supplemented with 10% fetal bovine serum, penicillin (100 U/ml) and streptomycin (100 μg/ml) (complete medium) at 37°C under humidified 5% CO_2_ conditions. After a 2-day culture period, the cells were provided with 2 ml of complete medium, and the medium was changed following another 2 days of culture. When the proliferating mouse embryo fibroblast cells (MEFs) reached confluence, they were suspended in medium supplemented with 10% dimethyl sulfoxide and stored in liquid nitrogen. The frozen stock of MEFs was thawed and allowed to grow for the subsequent experiments.

### X-irradiation

Exponentially growing cells were irradiated with X-rays using an X-ray generator (OM-B205; OHMic, Tokyo, Japan) operating at 70 kVp and 5 mA with a 0.5 mm Al filter at a dose rate of 0.64 Gy/min at room temperature.

### Combined treatment with AsA and X-irradiation

For use as a radical scavenger, AsA was dissolved in medium, adjusted to pH 7.2 and sterilized by a filter (pore size, 0.22 μm). For pre-treatment, the cells were incubated with the complete medium containing 5 mM AsA for 2 h before and during X-irradiation. After irradiation, the AsA-containing medium was changed to the complete medium and mitotic cells were harvested by colcemid (60 ng/ml) treatment for 3 h (2 h for unirradiated cells) for the preparation of chromosome samples. For post-treatment, the cells were incubated with the complete medium containing 5 mM AsA and colcemid (60 ng/ml) for 2 h after X-irradiation. The AsA-containing medium was removed, and the cells were incubated in the complete medium containing colcemid (60 ng/ml) for another 1 h (3 h in total) for the preparation of chromosome samples.

### Treatment with H_2_O_2_

Exponentially growing cells were treated with H_2_O_2_ at final concentrations of 100 and 200 μM for 1 h. After the removal of H_2_O_2_, the cells were washed with phosphate buffered saline (PBS) (−) and cultured in the complete medium containing colcemid (60 ng/ml) for 3 h for the harvesting of mitotic cells.

### Treatment with Aph

Exponentially growing cells were treated with Aph at a final concentration of 200 nM for 16 h. During the last 3 h, they were incubated in the complete medium containing Aph (200 nM) and colcemid (60 ng/ml) for the harvesting of mitotic cells.

### Combined treatment with Aph and X-irradiation

After irradiation with 0.4 Gy of X-rays, cells were treated with Aph at a final concentration of 5 nM for 16 h. During the last 3 h, they were incubated in the complete medium containing Aph (5 nM) and colcemid (60 ng/ml) for the harvesting of mitotic cells.

### Telomere FISH

For the preparation of chromosome samples, cells were treated with 0.075 M KCl for 40 min at room temperature and fixed in Carnoy’s fixative (methanol:acetic acid = 3:1) three times. Chromosomes were prepared for slide mounting using a metaphase spread apparatus (HANABI, ADscience Technologies, Funabashi, Japan). The chromosome slides were then immersed in PBS (−) at 37°C for 30 min, fixed in 4% formaldehyde in PBS (−) for 2 min and washed three times with PBS (−) for 5 min per time. The slides were treated with 1 mg/ml pepsin solution (pH 2.0) at 37°C for 1–4 min. After washing with PBS (−) for 15 s at 37°C, the slides were fixed in 4% formaldehyde in PBS (−) for 2 min, washed with PBS (−) three times for 5 min per time, then dehydrated through ethanol (70, 85 and 100%) at room temperature. Each slide was then coated with 10 μl of hybridization mixture, which consisted of 70% formamide, 1% (w/v) blocking reagent (Roche, Basel, Switzerland) in maleic acid buffer (pH 7.0) and 3 ng of fluorescence-labeled telomeric PNA probe [FITC-(CCCTAA)_3_, Biologica Co., Nagoya, Japan] and mounted with a coverslip (22 × 22 mm). The slides were then heated on an aluminum heat block at 80°C for 3 min and hybridized with the PNA probe for 5 h in a humidified dark box. After hybridization, the slides were washed twice in 70% formamide/10 mM Tris (pH 7.2) for 15 min, followed by washing with 50 mM Tris/150 mM NaCl (pH 7.5)/0.05% Tween 20 three times for 5 min each time. Finally, DNA was counterstained with 4′,6-diamidino-2-phenylindole (1.5 μg/ml, Vector Laboratories, Newark, CA), and chromosome samples were shielded with coverslips using nail polish. The chromosome samples were observed using fluorescence microscopes (Carl-Zeiss Microscopy, Tokyo; Olympus, Tokyo, Japan).

### Score of abnormal telomere FISH signals

**Fig. 1:**
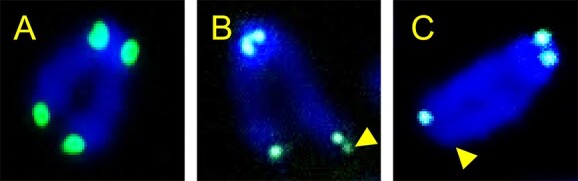
Abnormal telomere FISH signals. (**A**) Intact telomere signals that represent four dots of FISH signals at the tips of each chromosome arm. (**B**) An ETS, representing an extra dot at a tip of the chromosome arm (arrowhead). (**C**) An LTS, representing a missing dot at a tip of the chromosome arm (arrowhead).

A chromosome normally shows four dots of telomere FISH signals at the tips of each chromosome arm (Fig. 1A). Abnormal telomere FISH signals are categorized as either ETSs (Fig. 1B) or loss of telomere signals (LTSs; Fig. 1C). One extra dot signal was scored as one ETS, and two extra dot signals, although fewer in number, were scored as two ETS. Similarly, one missing dot signal was scored as one LTS, and two missing dot signals were scored as two LTS. Abnormal telomere FISH signals were quantified as the count of ETS or LTS per chromosome following the evaluation of 57–100 metaphases.

### Score of chromosome aberrations

Chromatid gaps, breaks and exchanges were scored as chromosome aberrations. One-hundred metaphases were examined.

## RESULTS

### Differential response to X-irradiation of two abnormal telomere FISH signals

**Fig. 2:**
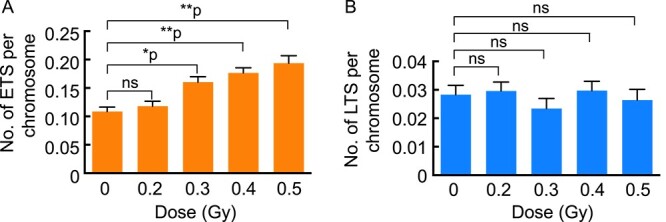
Induction of abnormal telomere FISH signals in MEFs by X-irradiation. The number of ETSs per chromosome and that of LTSs per chromosome in response to graded doses of X-rays is shown in (**A**) and (**B**), respectively. One-hundred cells were scored at each dose. Statistical analysis was performed using Dunn’s multiple comparisons test. ^*^P = 0.0008; ^*^^*^P < 0.0001; ns = not significant.

We examined the induction of abnormal telomere FISH signals with X-rays at low doses of 0.2–0.5 Gy. The induction responses differed between ETS and LTS, as shown in Fig. 2. The spontaneous induction level of ETS was 3.8-fold higher than that of LTS. The number of ETS increased dose-dependently from 0.3 to 0.5 Gy, although a significant induction was not seen at 0.2 Gy (Fig. 2A). We did not examine induction by higher doses because the induction of ETS was saturated at doses over 0.5 Gy. In contrast, LTS were not induced with X-rays at doses of 0.2–0.5 Gy (Fig. 2B). These results suggest that ETS, but not LTS, are generated as a consequence of telomere damage resulting from exposure to X-rays.

### The effect of AsA on the induction of abnormal telomere FISH signals and chromosome aberrations by X-irradiation

**Fig. 3:**
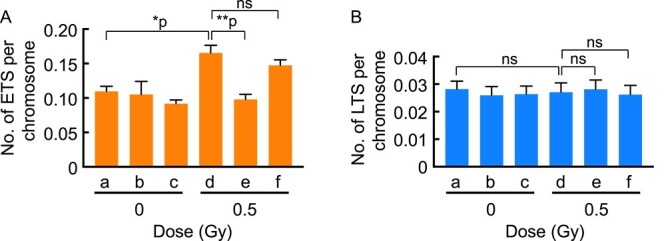
Effect of AsA on the induction of abnormal telomere FISH signals by X-irradiation. Counts of abnormal telomere signals (ETS, **A**; LTS, **B**) without (a–c) and with (d–f) combined treatment with AsA (5 mM, 2 h) and X-rays (0.5 Gy) are shown. (a) no treatment; (b) pre-treatment with AsA; (c) post-treatment with AsA; (d) X-rays; (e) X-rays plus pre-treatment with AsA; (f) X-rays plus post-treatment with AsA. One-hundred cells were scored at each treatment. Statistical analysis was performed using Dunn’s multiple comparisons test. ^*^P = 0.0031; ^*^^*^P < 0.0001; ns = not significant.

To clarify whether radicals are involved in X-ray-induced abnormal telomere FISH signals, we examined the effect of AsA as a radical scavenger on the induction of abnormal telomere FISH signals by X-irradiation. As shown in Fig. 3, pre-treatment (Fig. 3A-e), but not post-treatment (Fig. 3A-f), with AsA (5 mM, 2 h) suppressed X-ray-induced ETS (Fig. 3A-d) to a spontaneous level, although treatment with AsA itself did not change the ETS (Fig. 3A-b and c). In contrast, X-irradiation and any treatments with AsA did not change the induction of LTS (Fig. 3B). These results indicate the possibility that radicals scavenged by pre-treatment with AsA are responsible for the induction of ETS. However, the actual telomere damage that triggers the induction of ETS caused by those radicals remains obscure.

**Fig. 4:**
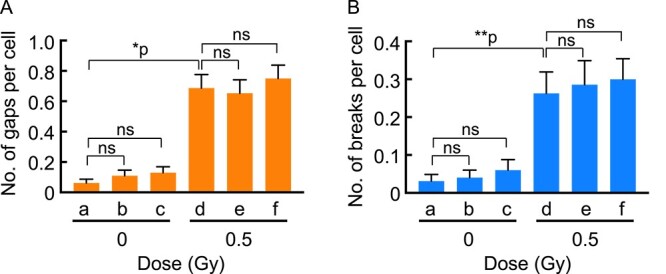
Effect of AsA on the induction of chromosome aberrations by X-irradiation. The counts of gaps per cell and breaks per cell without (a–c) and with (d–f) combined treatment with AsA (5 mM, 2 h) and X-rays (0.5 Gy) are shown in (**A**) and (**B**), respectively. (a) no treatment; (b) pre-treatment with AsA; (c) post-treatment with AsA; (d) X-rays; (e) X-rays plus pre-treatment with AsA; (f) X-rays plus post-treatment with AsA. The cell counts were as follows: (a) *n* = 97; (b) *n* = 100; (c) *n* = 100; (d) *n* = 99; (e) *n* = 98; (f) *n* = 100. Statistical analysis was performed using Dunn’s multiple comparisons test. ^*^P < 0.0001; ^*^^*^P = 0.0030; ns = not significant.

To explore the relationship between telomere damage, which leads to abnormal telomere FISH signals, and DNA damage, which results in chromosome aberrations, we investigated the induction of chromatid aberrations through combined X-ray treatment with AsA. X-rays (0.5 Gy) significantly induced chromatid gaps (Fig. 4A-d) and breaks (Fig. 4B-d), but not exchanges (data not shown). However, in contrast to the results observed with telomere signals, neither pre-treatment (Fig. 4A-e and B-e) nor post-treatment (Fig. 4A-f and B-f) with AsA (5 mM, 2 h) had any impact on the induction of chromatid gaps (Fig. 4A-d) or breaks (Fig. 4B-d) caused by X-irradiation.

These results suggest that the radicals removed by pre-treatment with AsA are responsible for ETS but are not involved in the induction of chromosome aberrations.

### Induction of abnormal telomere FISH signals by treatment with H_2_O_2_

**Fig. 5:**
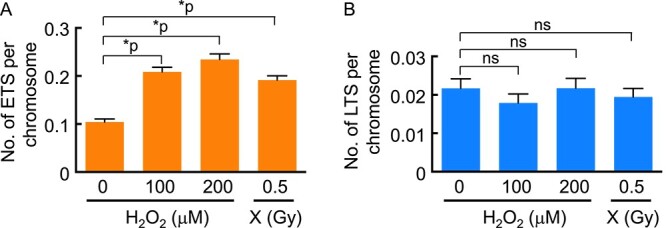
Induction of abnormal telomere FISH signals by treatment with H_2_O_2_. The ETS and LTS counts per chromosome in response to 100 and 200 μM H_2_O_2_ are shown in (**A**) and (**B**), respectively. X-rays (0.5 Gy) were used as a control for comparison. One-hundred cells were scored at each treatment. Statistical analysis was performed using Dunn’s multiple comparisons test. ^*^P < 0.0001; ns = not significant.

As our results suggested the involvement of X-ray-induced radicals in the induction of abnormal telomere FISH signals, we then examined the effect of treatment with H_2_O_2_, a reactive oxygen species (ROS), on the induction of ETS and LTS. As shown in Fig. 5, treatment with H_2_O_2_ (100 or 200 μM, 1 h) induced ETS (Fig. 5A), but not LTS (Fig. 5B), at a level equivalent to 0.5 Gy of X-rays (Fig. 5), suggesting that oxidative DNA damage is involved in the generation of ETS.

### Involvement of DNA replication stress in the induction of abnormal telomere FISH signals

**Fig. 6:**
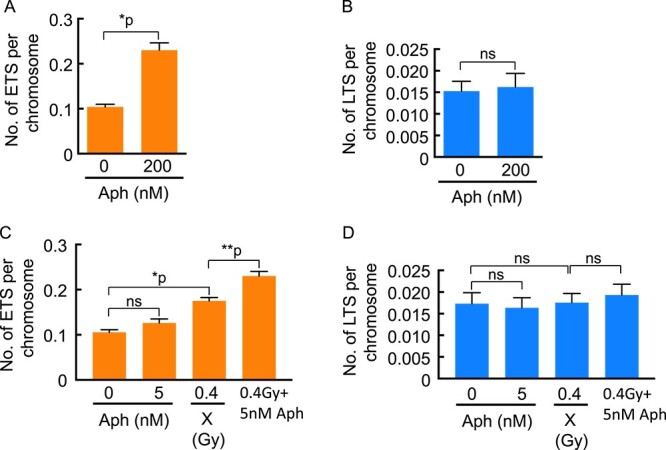
Effects of Aph and its combined treatment with X-rays on the induction of abnormal telomere FISH signals. The ETS and LTS counts per chromosome induced by Aph (Aph, 200 nM, 16 h) are shown in (**A**) and (**B**), respectively. The cell counts in A and B were as follows: no treatment, *n* = 100; 200 nM Aph, *n* = 57. Statistical analysis was performed using the Mann–Whitney test. ^*^P < 0.0001; ns, not significant. On the other hand, the ETS and LTS counts per chromosome induced by combined treatment with Aph (5 nM, 16 h) and X-rays (0.4 Gy) are shown in (**C**) and (**D**), respectively. One-hundred cells were scored at each treatment. Statistical analysis was performed using Dunn’s multiple comparisons test. ^*^P < 0.0001; ^*^^*^P = 0.0088; ns = not significant.

To clarify the causal effect of ETS generation by radical-related telomere damage, we examined the possibility that DNA replication stress may be involved in causing ETS by treating the cells with an inhibitor of DNA replication, Aph. The results indicated that treatment with 200 nM Aph significantly increased the number of ETS (Fig. 6A) but not LTS (Fig. 6B), suggesting that DNA replication stress is associated with the enhanced generation of ETS.

We then investigated the effect of combined treatment with Aph and X-irradiation on the induction of ETS and LTS. Because the combined treatment with 200 nM Aph and X-rays (0.4 Gy) induced cell-cycle arrest, we determined to reduce the Aph concentration to 5 nM, where treatment solely with it did not significantly induce ETS (Fig. 6C) or LTS (Fig. 6D). Intriguingly, the combined treatment with 5 nM Aph and 0.4 Gy of X-rays induced more ETS, but not LTS, than that by X-irradiation (0.4 Gy) alone. These results suggest that X-ray-induced telomere damage, which is assumed to be involved in ETS generation, is exacerbated due to the DNA replication stress by Aph.

## DISCUSSION

In the present study, we investigated the relationship between the induction of abnormal telomere signals detected by telomere FISH and exposure to X-rays or H_2_O_2_ and showed that oxidative DNA damage might be involved in the induction of signal abnormality. We previously reported that exposure to X-rays induced abnormal telomere FISH signals in human and rodent cells [10,13]. However, the doses (0.2–0.5 Gy) used in the present study (Fig. 2) are much lower than those used (>2 Gy) in the previous studies. The fact that abnormal telomere FISH signals increase dose-dependently at low doses (0.3–0.5 Gy) indicates that this abnormality is a radiation-sensitive biological response. Of particular interest is that pre-treatment, but not post-treatment, with AsA suppresses X-ray-induced ETS to the spontaneous level, indicating that radicals that can be scavenged by AsA play a role in inducing abnormal telomere FISH signals (ETS). This is the first report to show that scavenging X-ray-induced radicals results in a reduction of the abnormal FISH signals in telomeres.

It is noteworthy that, among ETS and LTS, only ETS increased in response to exposure to X-rays and H_2_O_2_. This implies that radical-mediated oxidative DNA damage may be a cause of ETS but not LTS. However, the telomere structure responsible for generating abnormal FISH signals, both ETS and LTS, remains to be determined. A chromosome break at the telomere, for instance, is not postulated to be a cause of abnormal telomere signals because AsA’s effect on chromosome gaps/breaks clearly differed from its effect on abnormal telomere FISH signals. If the emergence of ETS and LTS is associated with chromosome breaks at telomere regions, their responses to combined treatment with AsA and X-irradiation should be similar to those observed in chromosome aberrations. However, they were different, as shown in Figs 3 and 4. Rather, abnormal telomere signals may be caused by defects in telomere structures, including shelterin. Intriguingly, the appearance of similar abnormal telomere FISH signals was reported in *TRF1* null mouse cells, indicating that TRF1, a component of shelterin, is needed to maintain normal telomere FISH signals [14–16]. Indeed, the DNA binding ability of TRF1 and TRF2 proteins is inhibited by the presence of 8-oxoguanine (8-oxoG), a major oxidative DNA lesion [9]. Based on these observations, we propose that oxidative DNA damage initiates the emergence of abnormal telomere FISH signals, potentially serving as an indicator of a compromised protective structure for telomeres. So far, however, there is no direct evidence of a causal relationship between abnormal telomere FISH signals and telomere instability.

We previously reported that both pre-treatment and post-treatment with AsA (5 mM, 2 h) efficiently suppressed X-ray-induced gene mutation in human diploid cells; this observation suggests that the long-lived radicals, which are selectively scavenged by AsA, play a role in the induction of gene mutations [17]. However, the radicals involved in the induction of abnormal telomere FISH signals do not align with the long-lived radicals responsible for inducing gene mutations. This is because the suppressive effect by the post-treatment with AsA was evident only in the latter radicals. Interestingly, as evident in the present study, the previous study also showed that no change was observed in X-ray-induced chromosome aberrations by pre- and post-treatment with AsA (5 mM, 2 h) [17]. This finding suggests that the radicals effectively eliminated by AsA are not implicated in the development of X-ray-induced chromosome aberrations.

It is intriguing that the chronic, but not the acute, formation of telomeric 8-oxoG in human cancer cells lacking OGG1, the enzyme that removes 8-oxoG, triggers DNA replication stress at telomeres and increases telomere loss [18]. This implies that persistent oxidative DNA damage at telomeres plays a role in inducing replication stress, which drives telomere instability. In addition, the report by Coluzzi *et al*. demonstrates that DNA replication in telomeres is significantly reduced in human fibroblast cells treated with H_2_O_2_ (both 100 μM and 200 μM, 1 h) using the chromosome orientation-FISH technique [19]. These results indicate that the presence of oxidized DNA lesions within the telomeric repeats causes replication fork stall and suggest the possibility that this replication problem induces the emergence of abnormal telomere FISH signals. Furthermore, the results shown in Fig. 6 and reported by others using *TRF1* null mouse cells [15] indicate that the replication stress triggered by the Aph treatment elevates the abnormality of telomere FISH signals. Taking the previous and present results together, we propose that oxidative DNA lesions induce the replication stall in telomeres and that this replication stress is involved in the generation of abnormal telomere FISH signals, especially ETS.

In conclusion, we established that an ETS serves as a sensitive biological marker of oxidative DNA damage in telomere structures, including shelterin. Abnormal FISH signals might be responsible for some defects in telomere structures due to the generation of oxidative DNA damage. In future research, the structural defects responsible for abnormal telomere FISH signals must be explored.
